# CRISPR-Mediated Activation of αV Integrin Subtypes Promotes Neuronal Differentiation of Neuroblastoma Neuro2a Cells

**DOI:** 10.3389/fgeed.2022.846669

**Published:** 2022-04-12

**Authors:** Sara Riccardi, Lorenzo A. Cingolani, Fanny Jaudon

**Affiliations:** ^1^ Department of Life Sciences, University of Trieste, Trieste, Italy; ^2^ Department of Experimental Medicine, University of Genoa, Genoa, Italy; ^3^ Center for Synaptic Neuroscience and Technology (NSYN), Istituto Italiano di Tecnologia (IIT), Genoa, Italy; ^4^ IRCCS Ospedale Policlinico San Martino, Genoa, Italy

**Keywords:** integrins, CRISPRa, neurite outgrowth, neuronal differentiation, N2a cells

## Abstract

Neuronal differentiation is a complex process whose dysfunction can lead to brain disorders. The development of new tools to target specific steps in the neuronal differentiation process is of paramount importance for a better understanding of the molecular mechanisms involved, and ultimately for developing effective therapeutic strategies for neurodevelopmental disorders. Through their interactions with extracellular matrix proteins, the cell adhesion molecules of the integrin family play essential roles in the formation of functional neuronal circuits by regulating cell migration, neurite outgrowth, dendritic spine formation and synaptic plasticity. However, how different integrin receptors contribute to the successive phases of neuronal differentiation remains to be elucidated. Here, we implemented a CRISPR activation system to enhance the endogenous expression of specific integrin subunits in an *in vitro* model of neuronal differentiation, the murine neuroblastoma Neuro2a cell line. By combining CRISPR activation with morphological and RT-qPCR analyses, we show that integrins of the αV family are powerful inducers of neuronal differentiation. Further, we identify a subtype-specific role for αV integrins in controlling neurite outgrowth. While αVβ3 integrin initiates neuronal differentiation of Neuro2a cells under proliferative conditions, αVβ5 integrin appears responsible for promoting a complex arborization in cells already committed to differentiation. Interestingly, primary neurons exhibit a complementary expression pattern for β3 and β5 integrin subunits during development. Our findings reveal the existence of a developmental switch between αV integrin subtypes during differentiation and suggest that a timely controlled modulation of the expression of αV integrins by CRISPRa provides a means to promote neuronal differentiation.

## Introduction

Formation of functional neuronal circuits relies on the differentiation of neural stem cells and neural progenitors, a process involving cell cycle arrest and extension of neurites to generate axons and dendrites. Neuronal differentiation is tightly regulated during early brain development and persists, to a certain extent, in the adult nervous system, where it generates new neurons that integrate into existing neural networks under physiological conditions or upon injury (Gage and Temple, 2013; Ghosh, 2019; Denoth-Lippuner and Jessberger, 2021).

**FIGURE 1:**
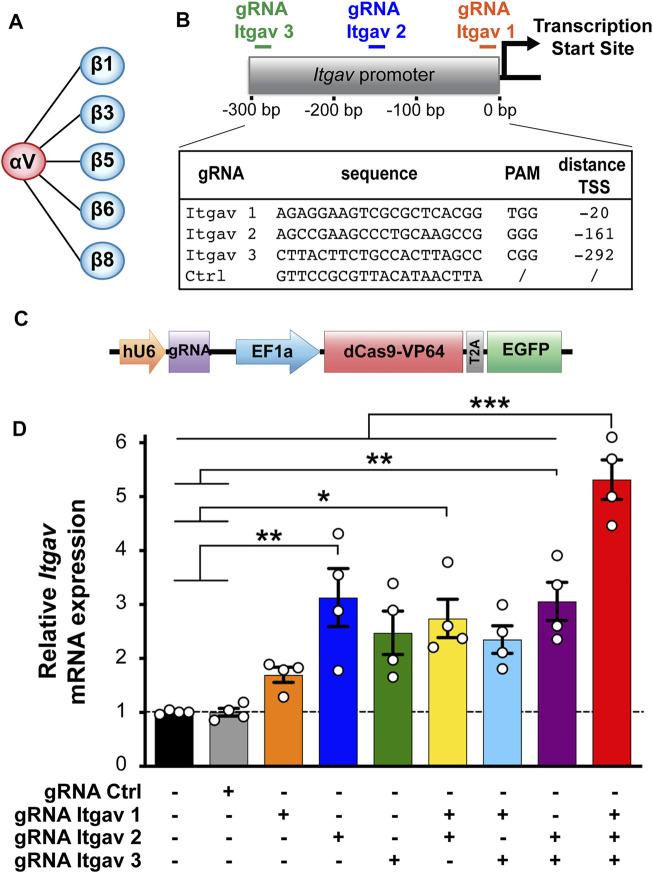
CRISPRa enhances expression of αV integrin subunit in N2a cells. **(A)** αV integrin receptor family. **(B)** gRNA sequences and position of their targets on the *Itgav* promoter. **(C)** Construct used for transfecting murine N2a cells, containing a cassette for expressing the gRNA and one for expressing dCas9-VP64 and EGFP. **(D)** Quantification of αV integrin mRNA levels in N2a cells 24 h after transfection with the indicated constructs. mRNA expression was normalized to the values of non-transfected samples within the same RT-qPCR plate. *p < 0.05, **p < 0.01, ***p < 0.001, one-way ANOVA followed by Tukey's post-test (*n* = 4 independent experiments).

By transducing chemical and mechanical signals from the extracellular matrix to the intracellular cytoskeleton, the cell adhesion molecules of the integrin family are key regulators of cell migration, neurite outgrowth, synaptogenesis and synaptic plasticity (Cheyuo et al., 2019; Chighizola et al., 2019; Jaudon et al., 2021; Lilja and Ivaska, 2018; Park and Goda, 2016; Prowse et al., 2011; Schmid and Anton, 2003; Thalhammer and Cingolani, 2014; Wojcik-Stanaszek et al., 2011). In mammals, there are 18 alpha and 8 beta subunits, forming 24 integrin heterodimers (Bachmann et al., 2019; Takada et al., 2007). How different integrin heterodimers cooperate in regulating neuronal differentiation remains, however, largely unknown. The αV subunit, which is highly expressed in both neural progenitor cells and neurons (Hall et al., 2006; Pinkstaff et al., 1999), dimerizes with five different beta subunits (β1, β3, β5, β6, and β8; Figure 1A) to form integrin receptors that recognize an RGD (arginine-glycine-aspartic acid) motif on their extracellular ligands. Previous studies have shown that αVβ3 integrin supports the proliferative capacity of neural progenitor cells (Fietz et al., 2010; Stenzel et al., 2014), while αVβ5 integrin promotes the differentiation of various types of neurons, such as cerebellar granule cells (Abe et al., 2018; Oishi et al., 2021) and retinal ganglion cells (Wang et al., 2006), suggesting a role for these integrins in regulating different aspects of neuronal differentiation.

Here, we investigate how enhancing the expression levels of two major αV integrin subunits (αV and β3) regulates neurite outgrowth and branching in an *in vitro* model of neuronal differentiation, the neuroblastoma Neuro2a (N2a) cell line. By combining the CRISPR activation (CRISPRa) technology, which relies on a catalytically inactive nuclease dead Cas9 (dCas9) fused to a transcriptional activator to enhance the expression of genes of interest (Gilbert et al., 2014), with morphological analyses and RT-qPCR, we show that αVβ3 integrin initiates differentiation of N2a cells under proliferative conditions, while αVβ5 integrin likely stabilizes neurites in N2a cells already committed to differentiation. We further show that β3 and β5 integrin subunits exhibit a complementary expression pattern during primary neuron development. Our findings reveal that neuronal differentiation requires a finely tuned division of labor between αV integrin subtypes.

## Materials and Methods

### gRNA Design and Plasmid Construction

We used the web tool http://crispr.mit.edu/ (Ran et al., 2013) to design three gRNAs targeting the region from −10 to −300 bp relative to the transcription start site (TSS) of the mouse *Itgav* gene. As negative control served a non-targeting gRNA sequence (gRNA Ctrl; Figure 1B). The gRNA sequences were inserted downstream of the U6 promoter into the pU6-(BbsI)-EF1a-dCas9-VP64-T2A-EGFP plasmid (Jaudon et al., 2019) using the BbsI cloning sites.

### N2a Cell Culture and Transfection

We cultured mouse neuroblastoma N2a cells in Dulbecco’s Modified Eagle Medium (DMEM, Gibco) supplemented with 10% FBS, 2 mM glutamine, 100 U/ml penicillin and 0.1 mg/ml streptomycin (complete culture medium), and maintained them in a 5% CO_2_ humidified incubator at 37°C. Cells were passaged 2–3 times a week at 80% confluence. For transfection, we seeded N2a cells in 6-well plates in complete culture medium at 200,000 and 100,000 cells/well for RT-qPCR and immunocytochemistry experiments, respectively. Coverslips were coated with 2.5 μg/ml poly-D-lysine (PDL; Cat. No. P7405, Sigma) or 5 μg/ml fibronectin (Cat. No. F8141, Sigma). The following day, cells were transfected with 4 μg DNA/well using the Ca^2+^ phosphate method (Thalhammer et al., 2017) and used for experiments 24–72 h post-transfection.

### N2a Cell Differentiation

Twenty-four hours after transfection, complete culture medium was replaced by serum-free medium (formulated as the complete medium but without FBS) to induce differentiation (Schubert et al., 1969). Under these conditions, neurite outgrowth was observed within 12–24 h. Differentiated cells were used for experiments 48 h after serum deprivation.

### Primary Cortical Culture

Cortical neuronal cultures were prepared from P0 C57BL/6J pups as previously described (Korotchenko et al., 2014; Thalhammer et al., 2017), with minor modifications. Briefly, cortices were dissected in ice-cold HBSS, digested with papain (30 U; Cat. No. 3126, Worthington) for 40 min at 37°C, washed and triturated in attachment medium (BME medium supplemented with 10% FBS, 3 mg/ml glucose, 1 mM sodium pyruvate and 10 mM HEPES-NaOH [pH 7.40]) with a flame-polished glass Pasteur pipette. Cells were seeded at a concentration of 95,000 cells/well onto 1.2 cm diameter glass coverslips coated with 2.5 μg/ml poly-D-lysine (PDL; P7405, Sigma) and 1 μg/ml laminin (L2020, Sigma). After 4 h, the attachment medium was replaced with maintenance medium (Neurobasal medium supplemented with 2.6% B27, 6 mg/ml glucose, 2 mM GlutaMax, 90 U/ml penicillin and 0.09 mg/ml streptomycin). To prevent glia overgrowth, 0.5 µM of cytosine β-D-arabinofuranoside (AraC) was added at 1 day *in vitro* (DIV).

### RNA Extraction and RT-qPCR

Total RNA was extracted with QIAzol lysis reagent (Cat. No. 79306, Qiagen) from transfected N2a cells or primary neurons at different developmental stages as previously described (Thalhammer et al., 2018). We prepared cDNAs by reverse transcription of 1 μg of RNA using the QuantiTect Reverse Transcription Kit (Cat. No. 205311, Qiagen). RT-qPCR was performed in triplicate with 10 ng of template cDNA using iQTM SYBR^®^ Green Supermix (Cat. No. 1708886, Biorad) on a CFX96 Real-Time PCR Detection System (Biorad) with the following universal conditions: 5 min at 95°C, 45 cycles of denaturation at 95°C for 15 s and annealing/extension at 60°C for 45 s. The relative quantification of gene expression was determined using the ∆∆Ct method. Data were normalized to glyceraldehyde-3-phosphate dehydrogenase (*Gapdh*), β-actin (*Actb*) and hypoxanthine phosphoribosyltransferase 1 (*Hprt1*) by the multiple internal control gene method with GeNorm algorithm (Vandesompele et al., 2002). Sequences of all the primers used are listed in Supplementary Table S1.

### Immunocytochemistry

Transfected cells were fixed for 10 min in 4% PFA at room temperature (RT), permeabilized for 10 min at RT with 0.2% Triton-X 100 and blocked for 30 min at RT with 5% NGS. Chicken anti-GFP (1:1,000; Cat. No. AB13970, Abcam) and rabbit anti-β tubulin III (1:500; Cat. No. T2200, Sigma-Aldrich) primary antibodies were used for either 2 h at RT or overnight at 4°C. Secondary antibodies were Alexa Fluor488-conjugated anti-chicken (1:1,000; Cat. No. A11039, ThermoFisher scientific) and Alexa Fluor568-conjugated anti-rabbit (1:1,000; Cat. No. A11036, ThermoFisher scientific). We stained cell nuclei with Hoechst (1 mg/ml; Cat. No. B2261, Sigma-Aldrich) and mounted coverslips with ProLong Gold (Cat. No. P10144, ThermoFisher scientific).

### Image Acquisition and Analysis

Three fields of view per coverslip were randomly selected and imaged using a Nikon Eclipse E800 epifluorescence microscope with a ×20 objective and a Nikon DXM1200 camera. Neurite length was measured in the red channel (β tubulin III) for all EGFP positive cells using the NeuronJ plugin of ImageJ (https://imagej.net/NeuronJ). Cells were considered differentiated if they had one or more processes at least twice as long as their cell bodies. We calculated the percentage of differentiated cells relative to the number of transfected cells (EGFP positive). Sholl analysis was performed on all differentiated transfected cells using the Sholl plugin of ImageJ (https://imagej.net/Sholl_Analysis) with a starting radius of 1 µm and a radius step size of 5 μm. For morphological analyses, we defined unipolar cells as those with only one unbranched process, bipolar cells as those with two unbranched processes extending in opposite directions from the soma and complex cells as those with more than two unbranched processes or displaying at least one neurite with branches.

### Statistical Analysis

**FIGURE 2:**
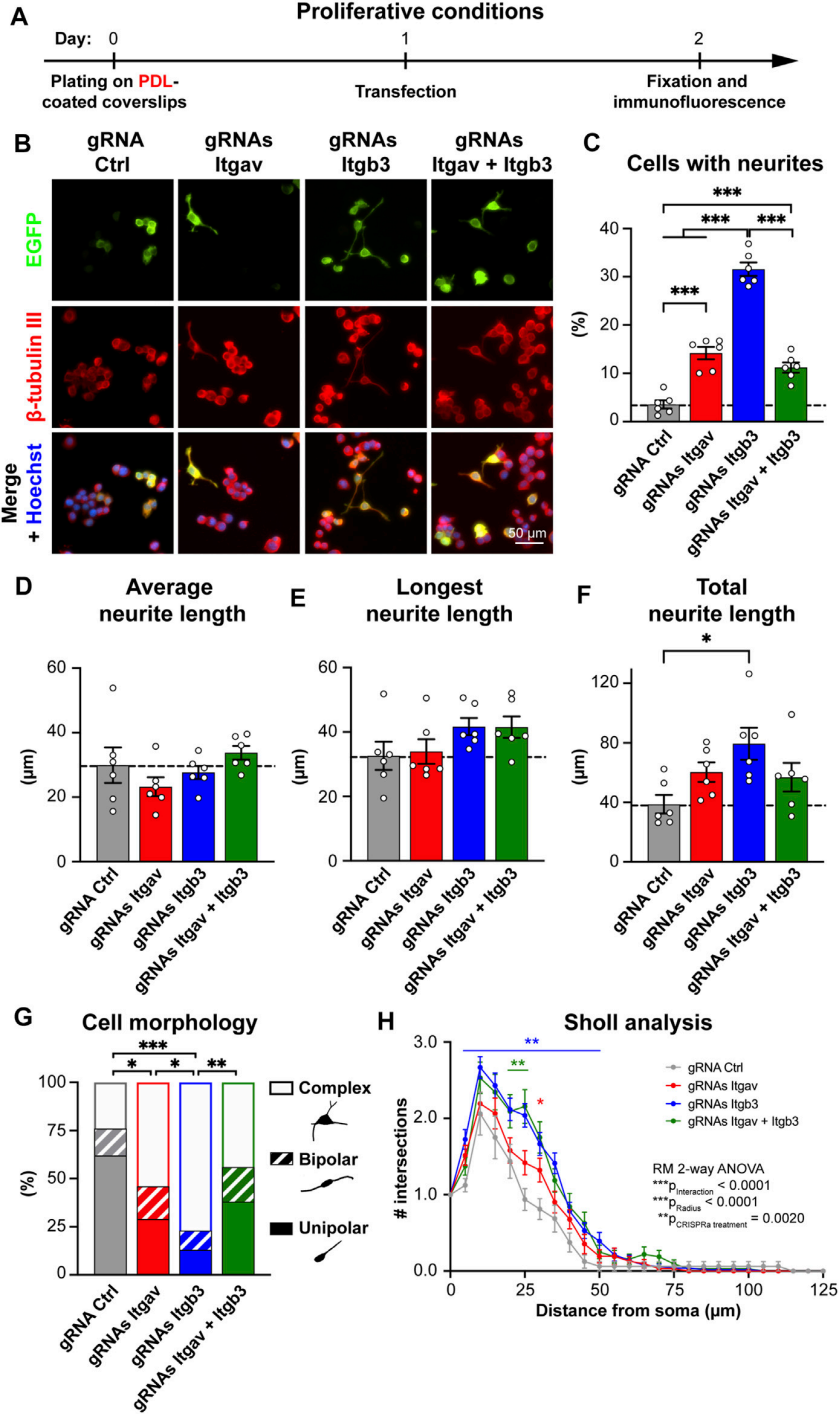
CRISPR-mediated activation of *Itgav* and *Itgb3* triggers N2a cell differentiation under proliferative conditions. **(A)** Time course of the experiment. **(B)** Representative images of N2a cells expressing the indicated constructs. Transfection was verified by EGFP expression, β-tubulin III staining was used to trace neurites and Hoechst to stain nuclei. **(C)** Percentage of cells with neurites within EGFP-positive cells for experiments as in **(A,B)**. ***p < 0.001, one-way ANOVA followed by Tukey’s post-test (*n* = 6 coverslips from four independent experiments). CRISPRa for either *Itgav* or *Itgb3* or both induces differentiation of N2a cells; CRISPRa for *Itgb3* alone is twice as effective as CRISPRa for *Itgav* alone or CRISPRa for *Itgav* and *Itgb3*. **(D–F)** Average **(D)**, longest **(E)** and total neurite length **(F)** of differentiated N2a cells expressing the indicated constructs. *p < 0.05, one-way ANOVA followed by Tukey’s post-test (*n* = 6 coverslips from four independent cultures). **(G)** Morphological classification of differentiated N2a cells. *p < 0.05, **p < 0.01, ***p < 0.001, Chi-square test (*n* = 17, 35, 51, and 34 cells from four independent experiments for gRNA Ctrl, gRNAs Itgav, gRNAs Itgb3 and gRNAs Itgav + Itgb3, respectively). **(H)** Sholl analysis of differentiated N2a cells. *p < 0.05 and **p < 0.01 relative to gRNA Ctrl, repeated measures ANOVA followed by Dunnett’s post-test (*n* = 17, 35, 51, and 34 cells from four independent experiments for gRNA Ctrl, gRNAs Itgav, gRNAs Itgb3 and gRNAs Itgav + Itgb3, respectively). CRISPRa for *Itgb3* induces a complex arborization.

**FIGURE 3:**
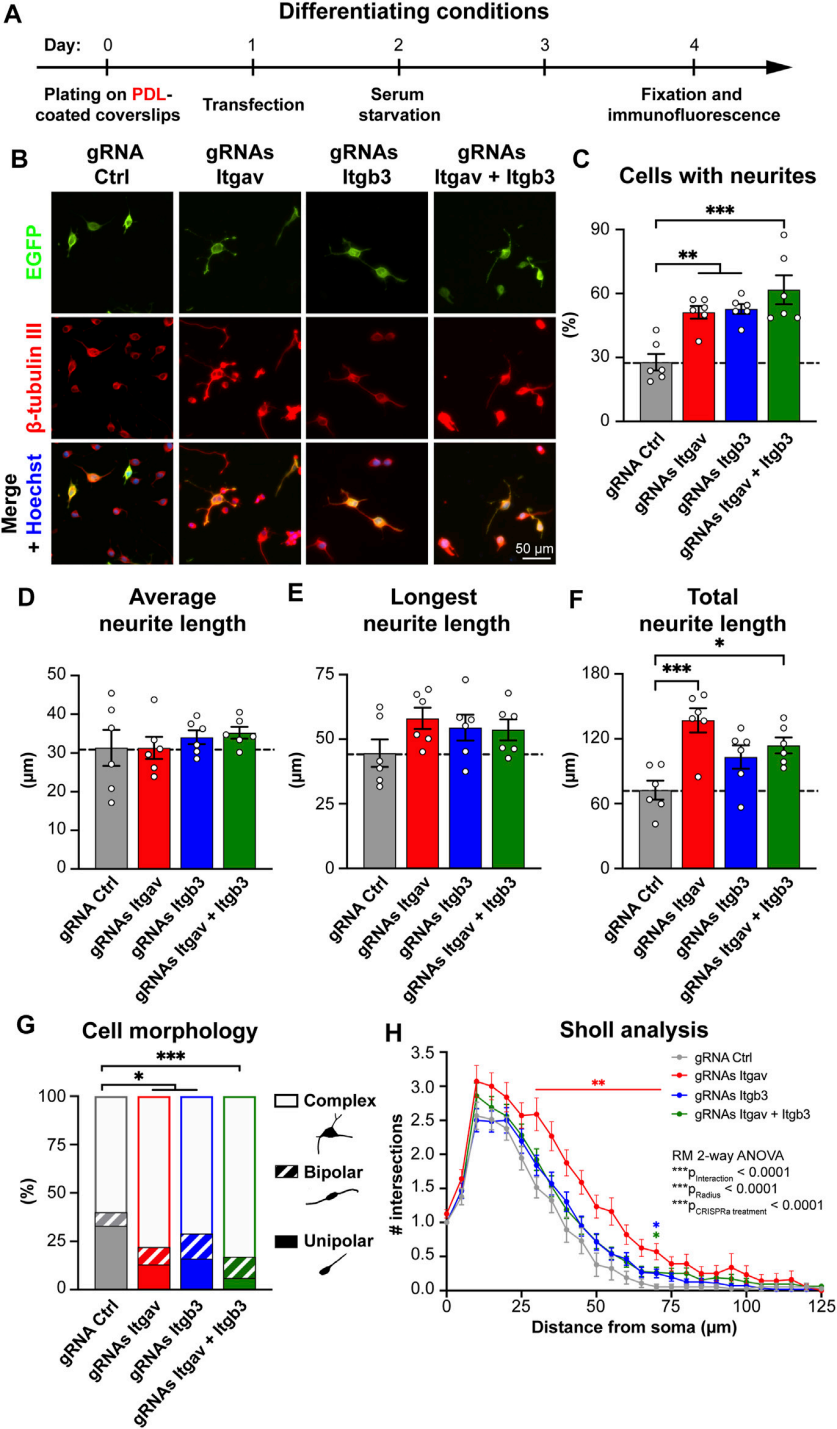
CRISPR-mediated activation of *Itgav* supports complex neurite arborization of differentiated N2a cells. **(A)** Time course of the experiment. **(B)** Representative images of N2a cells expressing the indicated constructs. Transfection was verified by EGFP expression, β-tubulin III staining was used to trace neurites and Hoechst to stain nuclei. **(C)** Percentage of cells with neurites within EGFP-positive cells for experiments as in **(A,B)**. **p < 0.01, ***p < 0.001, one-way ANOVA followed by Tukey’s post-test (*n* = 6 coverslips from 5 independent experiments). CRISPRa for either *Itgav* or *Itgb3* or both doubles the percentage of differentiated N2a cells, as compared to control conditions. **(D–F)** Average **(D)**, longest **(E)** and total neurite length **(F)** of differentiated N2a cells expressing the indicated constructs. *p < 0.05, ***p < 0.001, one-way ANOVA followed by Tukey’s post-test (*n* = 6 coverslips from 5 independent experiments). **(G)** Morphological classification of differentiated N2a cells. *p < 0.05, ***p < 0.001, Chi-square test (*n* = 55, 82, 75, and 83 cells from 5 independent experiments for gRNA Ctrl, gRNAs Itgav, gRNAs Itgb3 and gRNAs Itgav + Itgb3, respectively). **(H)** Sholl analysis of differentiated N2a cells. *p < 0.05, **p < 0.01 relative to gRNA Ctrl, repeated measures ANOVA followed by Dunnett’s post-test (*n* = 55, 82, 75, and 83 cells from 5 independent experiments for gRNA Ctrl, gRNAs Itgav, gRNAs Itgb3, and gRNAs Itgav + Itgb3, respectively). CRISPRa for *Itgav* induces a complex arborization.

Data are presented as mean ± SEM. Statistical significance was set at *p* < 0.05 and assessed using one-way ANOVA followed by the Tukey’s post hoc multiple comparison test, repeated measures two-way ANOVA followed by the Dunnett’s post hoc multiple comparison test or two-way ANOVA followed by the Tukey’s post hoc multiple comparison test, as specified in figure legends. The Chi-square test was used in Figures 2G, 3G; Supplementary Figures S1G, S2G (Prism 7, GraphPad Software, Inc.).

## Results

### CRISPRa Enhances αV Integrin Expression in N2a Cells

We have explored the possibility of using CRISPRa to enhance transcription of the αV integrin subunit as a means to promote neuronal differentiation of the murine neuroblastoma N2a cells. To this end, we have designed three gRNAs targeting the promoter of *Itgav* (the gene encoding αV integrin; Figure 1B) and expressed them in N2a cells together with dCas9 fused to the transcriptional activator VP64 (Perez-Pinera et al., 2013; Matharu et al., 2019). To minimize experimental variability, we have relied on a single vector designed to co-express a gRNA, dCas9-VP64 and the florescent protein EGFP (Figure 1C). EGFP allowed us to unambiguously identify transfected cells. As quantified by RT-qPCR 24 h post-transfection, all three gRNAs increased the expression of αV integrin by 2 to 3-fold, with gRNA Itgav-2 being the most effective (Figure 1D). We next combined the three gRNAs. While any combination of two of them did not further increase *Itgav* expression, combining all three of them increased it by 5.3-fold (Figure 1D). Thus, CRISPRa can be used to boost expression of endogenous αV integrin in N2a cells.

### αVβ3 Integrin Promotes Neuronal Differentiation of Proliferating N2a Cells

**FIGURE 4:**
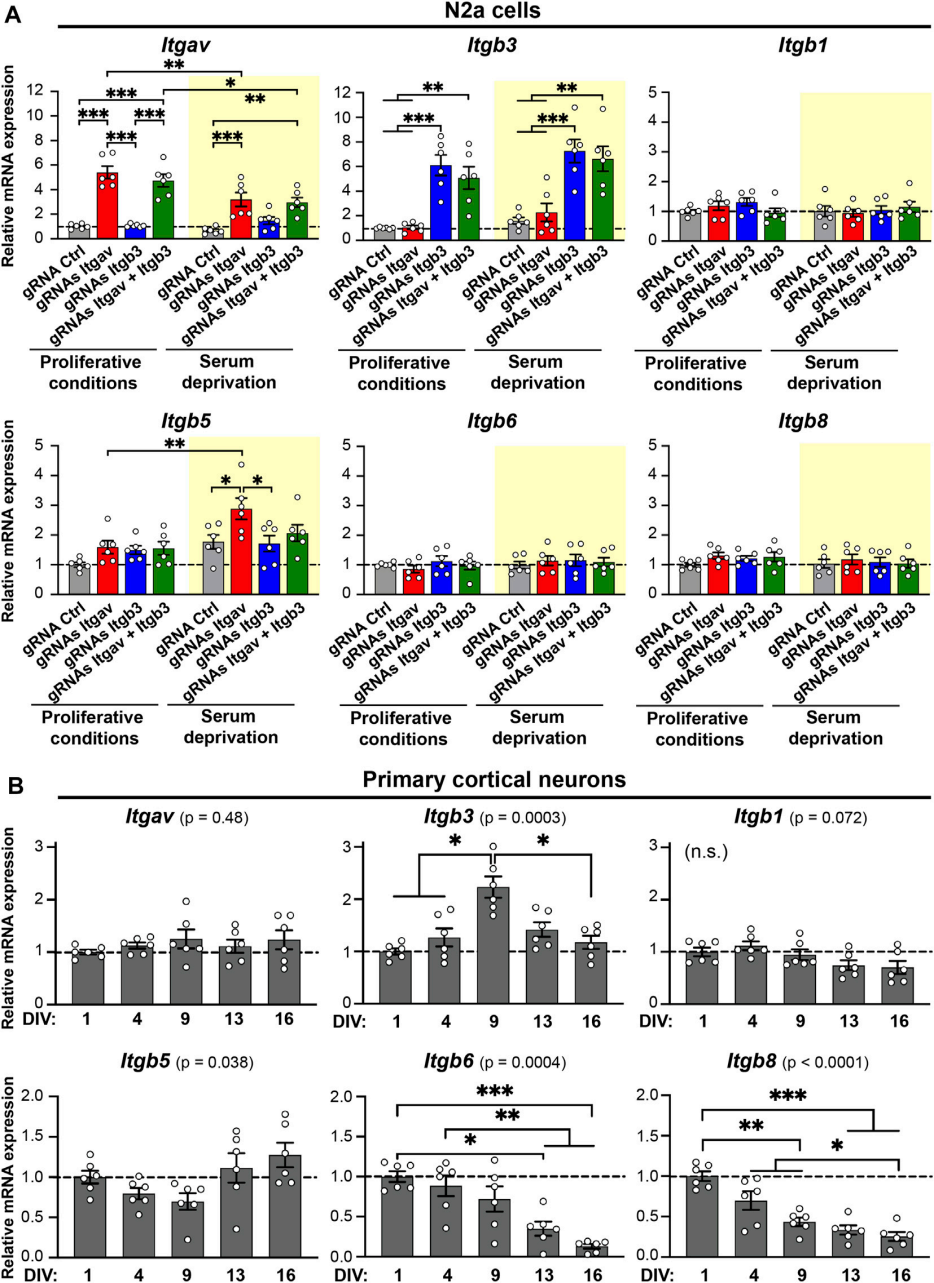
Expression levels of *Itgav*, *Itgb3*, *Itgb1*, *Itgb5*, *Itgb6*, and *Itgb8* in N2a cells and primary cortical neurons. **(A)** Quantification of *Itgav*, *Itgb3*, *Itgb1*, *Itgb5*, *Itgb6*, and *Itgb8* mRNA levels in response to CRISPRa for *Itgav* and/or *Itgb3* in N2a cells under proliferative conditions (white background) or following serum deprivation (yellow background). mRNA expression was normalized to the values of samples expressing gRNA Ctrl under proliferative conditions within the same RT-qPCR plate. *p < 0.05, **p < 0.01, ***p < 0.001, two-way ANOVA followed by Tukey's post-test (*n* = 6 from 3 independent cultures). CRISPRa for *Itgav* is accompanied by an increase in *Itgb5* expression levels in differentiating N2a cells. F ratio and *p* values for two-way ANOVA statistics are as follows, *Itgav:* gRNA effect: F_(3, 40)_ = 44.71, *p* < 0.001 (***); serum starvation effect: F_(1, 40)_ = 13.69, *p* < 0.001 (***); Serum starvation × gRNA interaction: F_(3, 40)_ = 5.351, *p* = 0.003 (**). *Itgb3:* gRNA effect: F_(3, 40)_ = 30.94, *p* < 0.001 (***); serum starvation effect: F_(1, 40)_ = 5.075, *p* = 0.03 (*); serum starvation × gRNA interaction: F_(3, 40)_ = 0.1581, *p* = 0.92. *Itgb1:* gRNA effect: F_(3, 40)_ = 0.5449, *p* = 0,65; serum starvation effect: F_(1, 40)_ = 0.6286, *p* = 0.43; serum starvation × gRNA interaction: F_(3, 40)_ = 1.189, *p* = 0.33. *Itgb5:* gRNA effect: F_(3, 40)_ = 4.606, *p* = 0.007 (**); serum starvation effect: F_(1, 40)_ = 17.01, *p* < 0.001 (***); serum starvation × gRNA interaction: F_(3, 40)_ = 1.818, *p* = 0.16. *Itgb6:* gRNA effect: F_(3, 40)_ = 0.4382, *p* = 0.7269; serum starvation effect: F_(1, 40)_ = 1.075, *p* = 0.3060; serum starvation × gRNA interaction: F_(3, 40)_ = 0.4069, *p* = 0.7488. *Itgb8:* gRNA effect: F_(3, 40)_ = 0.9408, *p* = 0.4300; serum starvation effect: F_(1, 40)_ = 1.302, *p* = 0.2606; serum starvation × gRNA interaction: F_(3, 40)_ = 0.3236, *p* = 0.8082. **(B)** Quantification of *Itgav*, *Itgb3*, *Itgb1*, *Itgb5*, *Itgb6*, and *Itgb8* mRNA levels in primary cortical neurons at different developmental stages. mRNA expression was normalized to the values of samples at 1 day *in vitro* (DIV) within the same RT-qPCR plate. *p < 0.05, **p < 0.01, ***p < 0.001, repeated-measures one-way ANOVA followed by Tukey's post-test (*n* = 6 from 3 independent cultures). F ratio and *p* values for repeated measures one-way ANOVA statistics are as follows, *Itgav:* F_(1.894, 9.469)_ = 0.7837, *p* = 0.4777. *Itgb3:* F_(2.643, 13.21)_ = 14.31, *p* = 0.0003 (***). *Itgb1:* F_(1.363, 6.813)_ = 4.258, *p* = 0.0723. *Itgb5:* F_(1.935, 9.676)_ = 4.698, *p* = 0.0384 (*). *Itgb6:* F_(2.051, 10.25)_ = 18.29, *p* = 0.0004 (***). *Itgb8:* F_(1.894, 9.471)_ = 39.24, *p* < 0.0001 (***).

We next addressed whether enhanced αV integrin expression promotes neuronal differentiation of N2a cells. When cultured in proliferative conditions (i.e., in medium containing 10% serum; Figures 2A,B), only 3.5% of cells expressing gRNA Ctrl exhibited at least one neurite. Maximal CRISPRa for αV integrin (using three gRNAs) induced neurite outgrowth in ∼14% of the cells (Figures 2B,C). We next compared these effects with those induced by CRISPRa for β3 integrin, a major partner of αV integrin in the brain (Figure 1A), and whose function has extensively been characterized in neurons in terms of synaptic function (Cingolani et al., 2008; Cingolani and Goda, 2008; McGeachie et al., 2011; McGeachie et al., 2012; Kerrisk et al., 2014; Park and Goda, 2016; Jaudon et al., 2021). To this end, we used two previously characterized gRNAs targeting the mouse promoter of *Itgb3*, the gene for β3 integrin. When used together, these two gRNAs increase β3 integrin expression in N2a cells by ∼6-fold (Jaudon et al., 2019); see also Figure 4A, top middle panel). Despite a similar efficacy of CRISPRa for the two integrin subunits, activation of *Itgb3* was twice as effective as activation of *Itgav* in inducing neurite outgrowth (∼32% and ∼14% of differentiated cells for *Itgb3* and *Itgav*, respectively; Figures 2B,C). We next co-activated *Itgav* and *Itgb3*. Co-activation was as effective as single gene activation in elevating *Itgb3* and *Itgav* expression (Figure 4A, top left and top middle panels), indicating that CRISPRa efficacy is not diluted by targeting two genes (with five constructs) instead of one (with two or three constructs). The effect on neurite outgrowth was nevertheless comparable to that obtained by activating *Itgav* alone (∼11% and ∼14% of differentiated cells for *Itgb3/Itgav* coactivation and *Itgav* activation, respectively; Figures 2B,C), indicating that the effects of αV integrin are dominant over those of β3 integrin.

Within the population of differentiated cells, increased expression of *Itgav* or *Itgb3* or both affected neither the average neurite length per cell (Figure 2D) nor the length of the longest neurite in each cell (Figure 2E). We detected only a significant increase in total neurite length per cell upon *Itgb3* activation (Figure 2F). Taken together, these results suggest that β3 integrin, rather than contributing to neurite elongation, promotes a higher number of processes and branches per cell. To verify this hypothesis, we next examined morphology and arborization of the differentiated cells by Sholl analysis. While differentiated cells expressing gRNA Ctrl were mainly unipolar, those transfected with gRNAs targeting *Itgav* or *Itgb3* or both presented predominantly a complex multipolar morphology with branched neurites. The effects were especially prominent when targeting exclusively *Itgb3* (Figure 2G). Accordingly, the Sholl analysis revealed that the cells with higher levels of β3 integrin displayed a more complex arborization than control cells (Figure 2H).

Because N2a cells were plated on a polycationic substrate (poly-D-lysine; PDL) that promotes cell adhesion in an integrin-independent manner, some of the differential effects between αV and β3 integrins on neurite outgrowth might be due to a limited availability of integrin subtype-specific extracellular ligands. To rule out this possibility, we next plated N2a cells on fibronectin, an extracellular matrix protein that binds to all αV-containing integrins (Johansson et al., 1997). Also under these experimental conditions, activation of *Itgb3* was more effective than that of *Itgav* in inducing neuronal differentiation and promoting a complex arborization (Supplementary Figure S1).

Because the β3 subunit pairs exclusively with the αV subunit in nucleated cells while the αV subunit pairs with five different beta subunits (Figure 1A; Hynes, 2002), these data suggest that *Itgb3* activation skews the composition of αV heterodimers towards αVβ3 integrin, thus promoting effectively neuronal differentiation of N2a cells maintained in proliferative conditions, while *Itgav* activation (alone or in combination with that of *Itgb3*) boosts the expression of αV heterodimers that restrain αVβ3 integrin function under proliferative conditions.

### αV Integrins Promote Neurite Arborization of Differentiated N2a Cells

In response to serum deprivation, N2a cells assume a morphology similar to that of mature neurons (Schubert et al., 1969). We therefore examined how CRISPRa for *Itgav* and *Itgb3* affects differentiation of N2a cells under pro-differentiating conditions (Figure 3A). As expected, removal of serum from the culture medium resulted in a higher percentage of differentiated cells in all conditions (Figures 2B,C vs. Figures 3B,C; for gRNA Ctrl: ∼28% vs. ∼3.5% of differentiated cells, respectively). CRISPRa for either *Itgav* or *Itgb3* or both effectively increased N2a differentiation also under these conditions. As opposed to the proliferative state, the effects of activating *Itgav* were however as robust as those induced by *Itgb3* activation (∼51% vs. ∼53% of differentiated cells, respectively). Moreover, co-activation of the two genes did not significantly change the percentage of differentiated cells as compared to single gene activation (Figures 3B,C; 62% of differentiated cells), suggesting either a shared mechanism involving up-regulation of αVβ3 integrin for the three experimental conditions or a concomitant contribution of different αV integrin-containing heterodimers to the differentiation process.

We next considered more closely the morphology of the differentiated cells. As in proliferative conditions, changes in the expression levels of *Itgav* or *Itgb3* or both affected neither the average neurite length per cell (Figure 3D) nor the length of the longest neurite in each cell (Figure 3E). As opposed to the proliferative conditions, the total neurite length per cell was, however, significantly increased upon *Itgav*, rather than *Itgb3*, activation (Figure 3F). Morphological and Sholl analyses further revealed that *Itgav* activation was more, rather than less, effective than *Itgb3* activation in promoting a complex arborization (Figures 3G,H). The differences between activation of *Itgav* and *Itgb3* for N2a cell differentiation were even more pronounced when the cells were plated on fibronectin (Supplementary Figure S2).

Thus, while αVβ3 integrin is extremely effective at inducing neuronal differentiation under proliferative conditions, other αV integrin-containing heterodimers appear to play a major role in stabilizing a complex arborization at later stages of differentiation.

### Expression of αV Integrin Subunits in N2a Cells and Primary Cortical Neurons

To get insights into which αV integrin heterodimers may favor branching and stabilization of neurites in N2a cells, we quantified, by RT-qPCR, the mRNA levels of all the beta subunits known to pair with the αV subunit (Figure 1A; Hynes, 2002; Lilja and Ivaska, 2018); under control conditions and following CRISPRa for *Itgav* or *Itgb3* or both (Figure 4A). As expected, targeting the promoter of *Itgav* and *Itgb3* with CRISPRa increased the expression level of the respective genes, both in proliferative and differentiating conditions (Figure 4A, top left and top middle panels). Strikingly, CRISPRa for *Itgav* increased also the expression of *Itgb5* (the gene for β5 integrin) selectively in differentiating conditions (∼63% increase relative to control differentiated conditions; Figure 4A, bottom left panel). Rather than being due to a direct effect of dCas9-VP64 on the *Itgb5* promoter, the increase in β5 integrin expression levels was likely the consequence of a co-regulation of *Itgav* and *Itgb5.* Indeed, no increase in β5 integrin expression levels was observed under proliferative conditions or under conditions favoring formation of the αVβ3 heterodimer (co-activation of *Itgav* and *Itgb3*; Figure 4A, bottom left panel).

Taken together, these findings suggest that up-regulation of αVβ5 integrin may promote branching and stabilization of neurites at later stages of differentiation in N2a cells.

To address whether the differences between αV integrin heterodimers are relevant in terms of neuronal differentiation, we next analyzed, in primary cortical neurons, the developmental expression pattern for all the subunits of αV integrin heteromers. Interestingly, the transcripts for *Itgb3* and *Itgb5* exhibited a complementary expression profile: *Itgb3* peaked at 9 DIV (Figure 4B, top middle panel), when dendritogenesis and synaptogenesis reach their maximum rate (Harrill et al., 2015), while *Itgb5* showed a dip during the same time period (Figure 4B, bottom left panel). The transcripts for *Itgav* and *Itgb1* remained stable from 1 to 16 DIV, while those for *Itgb6* and *Itgb8* decreased progressively during the same time period (Figure 4B). These data suggest therefore that the expression of αV integrins is dynamically regulated during neuronal differentiation.

## Discussion

Our findings indicate that CRISPRa for αV integrins promotes neuronal differentiation of neuroblastoma N2a cells, without the need for serum deprivation. Further, they strongly suggest a subunit-specific role of αV integrins in controlling neurite outgrowth. By combining CRISPRa, RT-qPCR and morphological analyses, we show that αVβ3 integrin initiates neuronal differentiation of N2a cells under proliferative conditions, while αVβ5 integrin is likely responsible for stabilizing neurites in cells already committed to differentiation.

Neuronal differentiation requires a series of consecutive steps starting with neural induction and cell proliferation events, followed by neuronal migration, neurite extension and axon-dendrite polarization. This precise differentiation program is due also to a coordinated rearrangement of the cytoskeleton in response to extracellular cues. Several integrins have been implicated in transducing extracellular signals to support outgrowth, branching or stabilization of neurites. For example, the laminin receptor α3β1 integrin signals through Arg kinase and p190RhoGAP to attenuate RhoA activity, thus stabilizing the dendritic arbor of adult, but not juvenile, hippocampal CA1 pyramidal neurons (Warren et al., 2012; Kerrisk et al., 2013), whereas β3 integrin contributes to establish a caudomedial-to-rostrolateral complexity gradient of basal dendrites in layer II/III cortical pyramidal neurons (Swinehart et al., 2020).

Integrins are also important for axon outgrowth. In the peripheral nervous system, α9β1 integrin functions as a receptor for the extracellular matrix protein tenascin-C, which is upregulated after injury, and indeed, exogenous expression of integrin α9 in dorsal root ganglia neurons promotes some axonal regeneration into the dorsal root entry after dorsal column crush lesion, resulting in limited sensory recovery (Andrews et al., 2009).

Comparatively less is known about how αV integrins, which are highly expressed in both neural progenitor cells and neurons (Pinkstaff et al., 1999; Hall et al., 2006), contribute to neurite outgrowth at early stages of differentiation. This group of integrins comprise five members (Figure 1A), whose expression is finely modulated during maturation of cortical neurons (Figure 4B).

To decipher their role in the initial steps of neurite outgrowth, we induced a 5 to 6-fold increase in the endogenous expression of αV or β3 integrin subunits or both in N2a cells maintained in proliferative conditions. While both subunits promoted neuronal differentiation of N2a cells, we observed a stronger induction with β3 integrin. Given that this subunit pairs exclusively with the αV in nucleated cells (Hynes, 2002; Lilja and Ivaska, 2018), an increase in β3 integrin expression levels is likely to shift the balance of all αV-containing receptors towards the αVβ3 heterodimer. This αV integrin appears therefore the most effective in promoting extension and branching of neurites in the initial phases of N2a cell differentiation.

The situation was surprisingly reversed in N2a cells already committed to neuronal differentiation by serum deprivation: neurite complexity was supported more effectively by activation of the αV rather than the β3 subunit. Because boosting αV expression in differentiated cells induced also a concomitant upregulation of the β5 subunit, we concluded that the αVβ5 heterodimer is likely the most important αV integrin in stabilizing neurites at later stages of differentiation.

This shift in dependency for neurite arborization from αVβ3 to αVβ5 integrin likely reflects differences in subcellular localization or signalling between the two integrins (Baschieri et al., 2018; Lock et al., 2018; Zuidema et al., 2018), rather than a limited access to extracellular ligands. Indeed, both αVβ3 and αVβ5 integrins exhibit robust cell surface expression without the need for specific extracellular ligands (Sun et al., 2021), and the differences in neurite differentiation between the two heterodimers were observed irrespective of the plating substrate. The most prominent effect of fibronectin was actually to inhibit neurite outgrowth following CRISPRa for *Itgav* under proliferative conditions; in comparison, there were only minor effects of fibronectin upon CRISPRa for *Itgb3* or *Itgb3* and *Itgav* under the same proliferative conditions (compare Figure 2C with Supplementary Figure S1C).

Recent data show that αVβ3 integrin is able to bind fibronectin only in an extended-open conformation following high mechanical load (Bachmann et al., 2020; Jaudon et al., 2021), which may favour dynamic binding-unbinding to and from the substrate during early neuritogenesis. Indeed, in many cell types, αVβ3 integrin localizes in highly dynamic focal adhesion complexes linked to talin and actin stress fibers, while αVβ5 integrin has recently been found enriched in a new type of adhesion complex (referred to as flat clathrin lattices), which lacks classical adhesion proteins and associates with branched cortical actin, rather than actin stress fibers (Lock et al., 2018; Zuidema et al., 2018). Despite being rich in components of the clathrin-mediated endocytosis machinery, flat clathrin lattices are highly static structures with very low endocytic activity. This is mainly due to αVβ5 integrin binding tightly to the substrate, thus preventing the formation of clathrin-coated pits (Baschieri et al., 2018). These unique features of αVβ5 integrin are most likely due to an insert of eight amino acids in the cytosolic tail of the β5 subunit, which is not found in any other beta subunit and may be responsible for the subcellular localization of αVβ5 integrin in flat clathrin lattices (Zuidema et al., 2018). Although these adhesion complexes have not been described in N2a cells and neurons, it is possible that αVβ5 integrin supports more static cell-substrate interactions that slow down outgrowth of neurites at early stages of neuronal differentiation, while effectively stabilizing them once formed.

Our results on the cooperation between αV integrin heterodimers in regulating different stages of N2a cell differentiation are in line with previous findings in granule cell precursors of the developing cerebellum. In these cells, αVβ3 integrin contributes to suppressing proliferation, whereas αVβ5 integrin promotes the transition to granule cells and induces axon specification *via* a signalling pathway involving the kinases PI3K, Akt and GSK3β (Abe et al., 2018; Oishi et al., 2021). Therefore, a shift from αVβ3 integrin- to αVβ5 integrin-mediated cell adhesion might represent a general mechanism during development for promoting the transition from proliferating precursors to differentiating cells.

Here, we showed that CRISPRa can be used to regulate effectively the expression of these integrins, thereby promoting a coordinated progression of N2a cells towards a neuron-like phenotype. CRISPRa is not as prone as CRISPR-mediated genome editing to off-target effects because it does not rely on genomic DNA cleavage but requires persistent binding to a promoter region. Furthermore, the possibility of targeting one or multiple genes, using one or multiple gRNAs for each gene, and the availability of various transcriptional activators, such as VP64, VPR, SAM, and Suntag (Chavez et al., 2015; Konermann et al., 2015), as well as transcriptional repressors for CRISPR interference (CRISPRi), makes this system easily customable to regulate precisely gene expression (Zheng et al., 2018; Zhou et al., 2018; Matharu et al., 2019; Savell et al., 2019). By contrast, overexpression paradigms are far less versatile and elevate the expression of the gene of interest by several folds with possible toxic effects (Jaudon et al., 2019). A temporally controlled activation of gene expression could be achieved by combining CRISPRa with inducible systems such as the tetracycline-dependent promoter (Tet) system (Dow et al., 2015; de Solis et al., 2016) or photoactivable proteins (Kawano et al., 2015). Thus, it could be possible to switch the expression of distinct integrin subunits at specific steps of neuronal differentiation.

In summary, our findings show that CRISPRa-mediated enhancement of αV integrins is a powerful and versatile strategy to induce neurite outgrowth and stabilization. These new tools could be used to promote neuronal differentiation of induced pluripotent stem cells, which express αV integrins (Rowland et al., 2010), or to ameliorate neurite abnormalities in mouse models of brain disorders.

## Data Availability

The original contributions presented in the study are included in the article/Supplementary Material, further inquiries can be directed to the corresponding authors.
