# Supplementary material for: CRISPR-Mediated Activation of αV Integrin Subtypes Promotes Neuronal Differentiation of Neuroblastoma Neuro2a Cells
Source: Front Genome Ed. 2022 Apr 12;4:846669. doi: 10.3389/fgeed.2022.846669 (PMC9039181; doi:10.3389/fgeed.2022.846669)
Supplement: Supplementary file 1 [file DataSheet1.PDF]

Supplementary Material

CRISPR-mediated activation of αV integrin subtypes promotes neuronal differentiation of neuroblastoma Neuro2a cells

Sara Riccardi[1,2], Lorenzo A. Cingolani[1,3] and Fanny Jaudon[1,4]

[1]Department of Life Sciences, University of Trieste, Trieste, Italy

[2]Department of Experimental Medicine, University of Genoa, Genoa, Italy

[3]Center for Synaptic Neuroscience and Technology (NSYN), Istituto Italiano di Tecnologia (IIT), Genoa, Italy

[4]IRCCS Ospedale Policlinico San Martino, Genoa, Italy

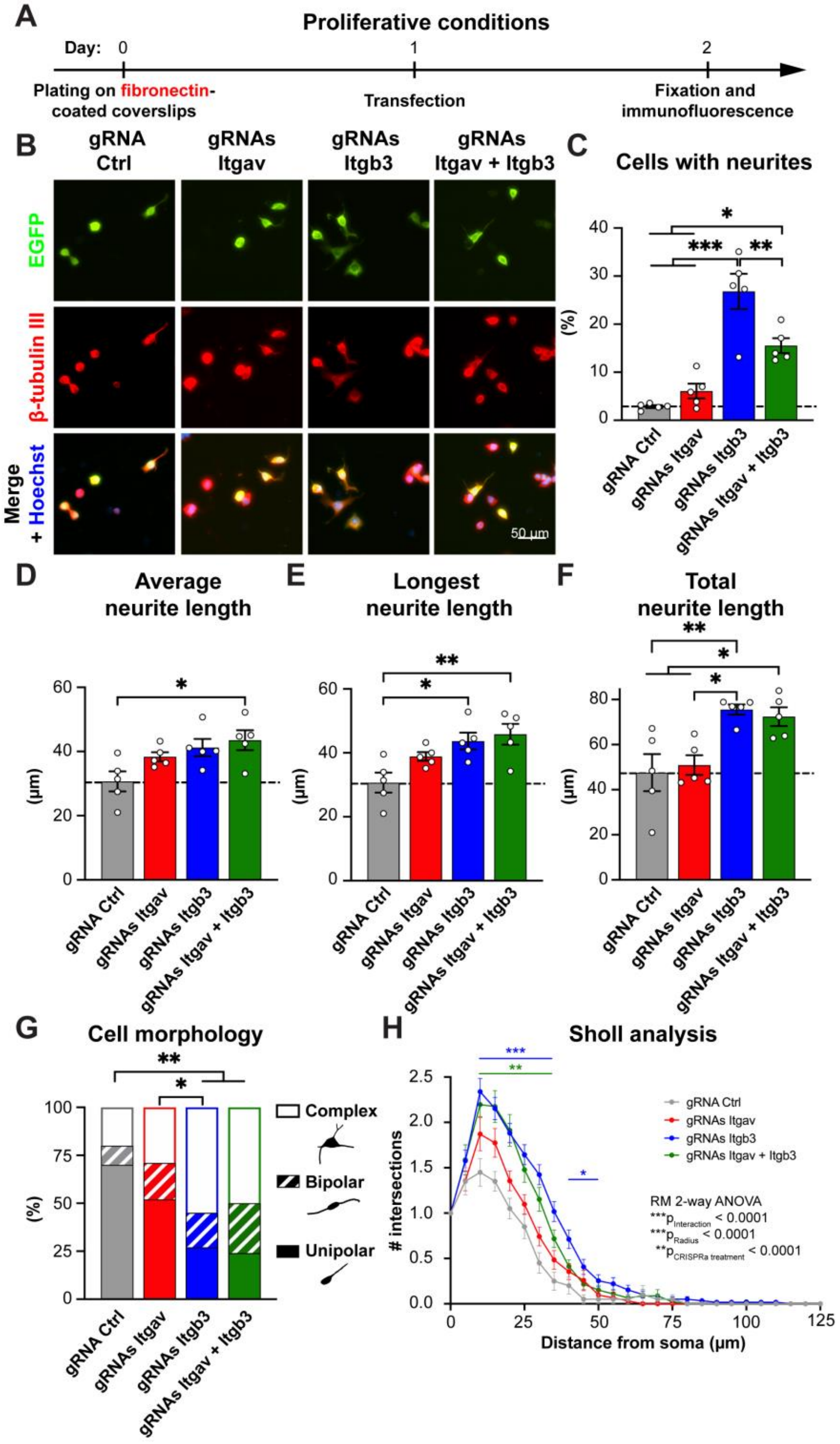

Supplementary Figure 1. Differentiation of N2a cells plated on fibronectin under proliferative conditions upon CRISPRa for *Itgav* and/or *Itgb3*. (A) Time course of the experiment. **(B)** Representative images of N2a cells expressing the indicated constructs. Transfection was verified by EGFP expression, β-tubulin III staining was used to trace neurites and Hoechst to stain nuclei. **(C)** Percentage of cells with neurites within EGFP-positive cells for experiments as in (A, B). *$p<0.05$, **$p<0.01$, ***$p<0.001$ one-way ANOVA followed by Tukey's post-test (n=5 coverslips from 3

independent experiments). CRISPRa for *Itgb3* induces differentiation of N2a cells. **(D-F)** Average (D), longest (E) and total neurite length (F) of differentiated N2a cells expressing the indicated constructs. *p<0.05, **p<0.01, one-way ANOVA followed by Tukey's post-test (n=5 coverslips from 3 independent cultures). **(G)** Morphological classification of differentiated N2a cells. *p<0.05, **p<0.01, Chi-square test (n=20, 31, 59 and 46 cells from 3 independent experiments for gRNA Ctrl, gRNAs Itgav, gRNAs Itgb3 and gRNAs Itgav + Itgb3, respectively). **(H)** Sholl analysis of differentiated N2a cells. *p<0.05, **p<0.01, ***p<0.001 relative to gRNA Ctrl, repeated measures ANOVA followed by Dunnett's post-test (n=20, 31, 59 and 46 cells from 3 independent experiments for gRNA Ctrl, gRNAs Itgav, gRNAs Itgb3 and gRNAs Itgav + Itgb3, respectively). CRISPRa for *Itgb3* induces a complex arborization.

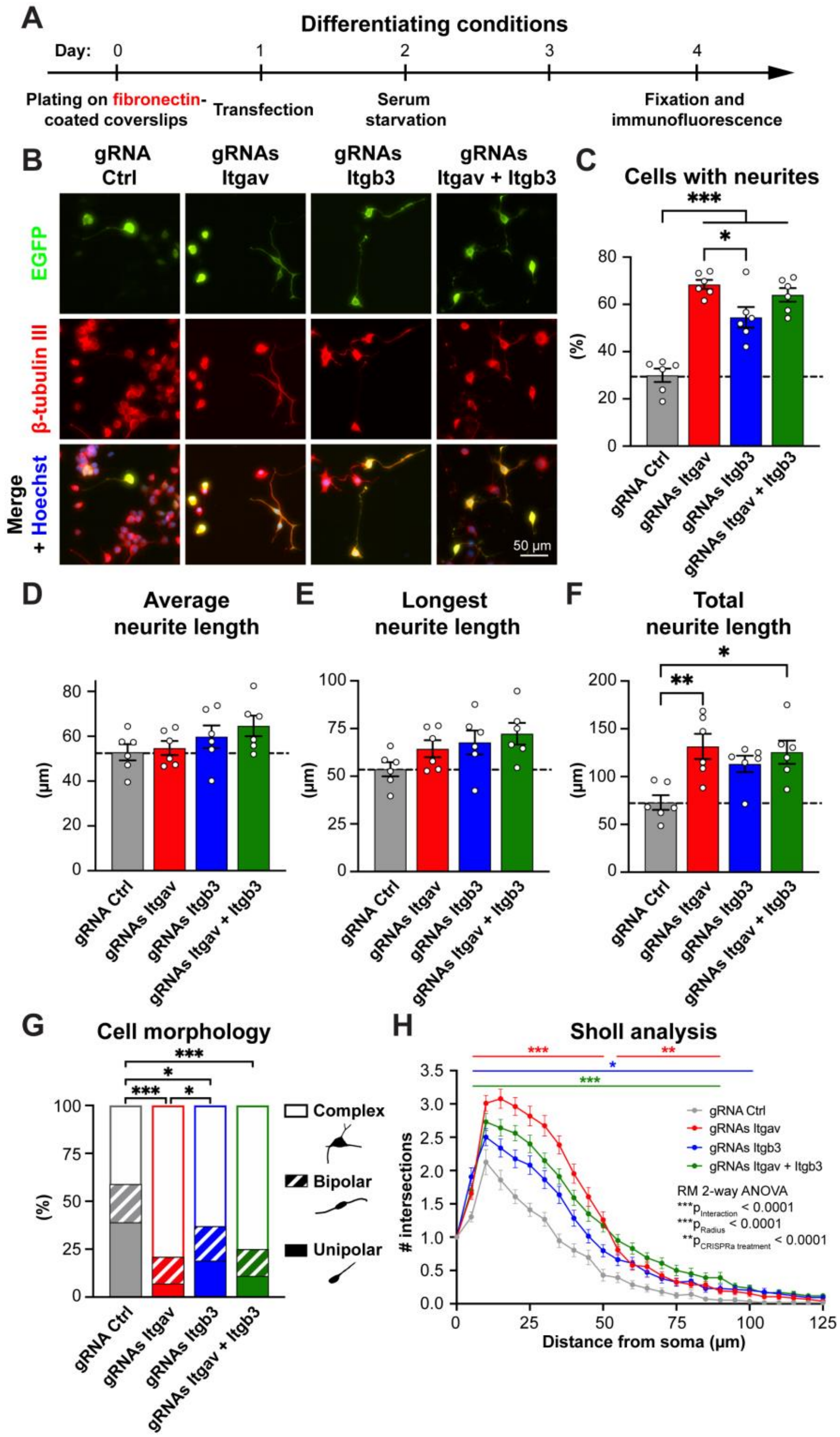

Supplementary Figure 2. Differentiation of N2a cells plated on fibronectin under differentiating conditions upon CRISPRa for *Itgav* and/or *Itgb3*. (A) Time course of the experiment. **(B)** Representative images of N2a cells expressing the indicated constructs. Transfection was verified by EGFP expression, β-tubulin III staining was used to trace neurites and Hoechst to stain nuclei. **(C)** Percentage of cells with neurites within EGFP-positive cells for experiments as in (A, B). *p<0.05, ***p<0.001, one-way ANOVA followed by Tukey's post-test (n=6 coverslips from 3 independent

experiments). CRISPRa for either *Itgav* or *Itgb3* or both doubles the percentage of differentiated N2a cells, as compared to control conditions. **(D-F)** Average (C), longest (D) and total neurite length (E) of differentiated N2a cells expressing the indicated constructs. *p<0.05, **p<0.01, one-way ANOVA followed by Tukey's post-test (n=6 coverslips from 3 independent experiments). **(G)** Morphological classification of differentiated N2a cells. *p<0.05, ***p<0.001, Chi-square test (n=56, 104, 74 and 100 cells from 3 independent experiments for gRNA Ctrl, gRNAs Itgav, gRNAs Itgb3 and gRNAs Itgav + Itgb3, respectively). **(H)** Sholl analysis of differentiated N2a cells. *p<0.05, **p<0.01, ***p<0.001 relative to gRNA Ctrl, repeated measures ANOVA followed by Dunnett's post-test (n=56, 104, 74 and 100 cells from 3 independent experiments for gRNA Ctrl, gRNAs Itgav, gRNAs Itgb3 and gRNAs Itgav + Itgb3, respectively). CRISPRa for *Itgav* induces a complex arborization.

Supplementary Table 1. List of RT-qPCR primers used.

Gene	GenBank Accession	Forward sequence (5' → 3')	Reverse sequence (5' → 3')
Itgav	NM_008402.3	ATTGACGGGCCAATGAACTG	ATTCCACAGCCCAAAGTGTG
Itgb1	NM_010578.2	CTTATTGGCCTTGCCTTGCT	GATTTTCACCCGTGTCCCAC
Itgb3	NM_016780.2	GGGCGTTGTTGTTGGAGAG	ACAAAGTCTCATCTGAGCACCAG
Itgb5	NM_001145884.1	CCGTGAGCCGGAGCG	GCATATGTTGAGCCCTGCG
Itgb6	NM_001159564.1	ATCATGTTGGGGGTGTCACT	ACAGAGGATTGGTTCCCGTT
Itgb8	NM_177290.3	CTGCCGTCTGTGAGAGTCAT	CGTCATTCGGCACCACTATG
Actb	NM_007393.5	TTGCTGACAGGATGCAGAAG	AGTCCGCCTAGAAGCACTTG
Gapdh	NM_001289726.1	TGTGTCCGTCGTGGATCTGA	CCTGCTTCACCACCTTCTTGA
Hprt1	NM_013556.2	AAGCTTGCTGGTGAAAAGGA	TTGCGCTCATCTTAGGCTTT
